# Human genetic deficits in glycan formation

**Published:** 2004-03-01

**Authors:** Tamao Endo

**Affiliations:** Glycobiology Research Group, Tokyo Metropolitan Institute of Gerontology, Foundation for Research on Aging and Promotion of Human Welfare, 35-2, Sakaecho, Itabashi-ku, Tokyo 173-0015

**Keywords:** *O*-mannosylation, muscular dystrophy, glycosyltransferase, congenital disorder of glycosylation, *N*-glycosylation

## Abstract

Glycans are associated with most proteins found in secretions and on the surface of mammalian cells. Glycans of secreted glycoproteins affect many protein properties such as solubility, stability, protease sensitivity, and polarity, while glycans on cell surface glycoproteins are involved in various cellular functions including cell-cell and cell-matrix interactions during embryogenesis, immune reactions, and tumor development. Recent advances in human genomic research together with newly developed and sensitive methods for the analysis of glycan structures have elucidated the etiology of a growing number of human genetic diseases with aberrant glycan formation. Among these diseases, defects of protein *N*-glycosylation and *O*-mannosylation are reviewed here. The former is relatively common and the latter is rather uncommon. Both types of defects lead to severe abnormalities, which indicate the importance of glycosylation. Sequencing of the human genome is essentially complete and now glycobiology becomes an important area of postgenomic research. Glycobiology is expected to produce remarkable advances in the understanding and treatment of certain genetic diseases.

## Introduction

Recent advances in glycobiology have revealed the importance of sugar chains as biosignals for multi-cellular organisms including cell-cell adhesion, cell-matrix adhesion, extracellular receptor-ligand interactions, quality control of proteins, and sorting of proteins within cells, and regulation of intracellular signal transduction processes.1) These studies have elucidated the regulation of various fundamental biological processes, including cell migration, cell fate determination, and morphogenesis, and mechanisms that modulate development. Because over 60% of the proteins produced by the human body are thought to contain sugar chains, a large number of important physiological events are possibly related to the research field of glycobiology. The major sugar chains of glycoproteins can be classified into two groups according to their sugar-peptide linkages. Those linked to asparagine (Asn) residues of proteins are termed *N*-glycans, while those linked to serine (Ser) or threonine (Thr) residues are called *O*-glycans. In *N*-glycans, the reducing terminal *N-*acetylglucosamine (GlcNAc) is linked to the amide group of Asn via an aspartylglycosylamine linkage. In *O-*glycans, the reducing terminal *N*-acetylgalactosamine (GalNAc) is attached to the hydroxyl group of Ser and Thr residues. In addition to the abundant *O*-GalNAc forms, several unique types of protein *O*-glycosylation have been found, such as *O*-linked fucose, glucose, GlcNAc, and mannose, which have been shown to mediate diverse physiological functions. For example, *O-*fucose has been identified on epidermal growth factor-like repeats for Notch, and elongation of *O*-fucose has been implicated in the modulation of Notch signaling by Fringe.2)–5)

The biosynthesis of sugar chains is not controlled by the intervention of a template, and the sugar chains are formed as secondary gene products by the concerted action of glycosyltransferases. There is growing evidence that these enzymes are involved in cellular differentiation and development, and disease processes. The removal of glycosyltransferase genes in knockout mice indicates that some glycosyltransferases are essential for development, and their defects lead to abnormalities.6) The importance of sugar chains is further highlighted by congenital disorders of glycosylation (CDGs, which are caused by defects in *N*-glycans) that result in hypotonia, psychomotor retardation, coagulopathies, and gastrointestinal signs and symptoms. Sugar chains other than those of the *N*-linked pathway are also important. This is demonstrated by the finding that aberrant *O*-mannosylation is the primary cause of some forms of congenital muscular dystrophy with abnormal neuronal migration. Protein *N*-glycosylation is a common modification, while *O*-mannosylation is an unusual type of protein modification.7) Defects of both glycosylation lead to severe abnormalities, indicating that glycosylation is important. This article reviews new insights into glycobiology of human glycan abnormality.

## *N*-Glycan formation and congenital disorders of glycosylation (CDG)

*O*-Glycans are formed by stepwise addition of monosaccharides to the Ser and Thr residues of polypeptides from nucleotide sugars. In contrast, *N*-glycans are formed by a series of complex pathways including lipid-linked intermediates. First, GlcNAc-1-P is transferred from UDP-GlcNAc to a polyisoprenol monophosphate: dolichyl phosphate (Dol-P). The GlcNAc residue of the GlcNAc-PP-Dol is the starting point of *N*-glycans. To this GlcNAc residue, another GlcNAc and five mannose residues are transferred from UDP-GlcNAc and GDP-Man, respectively. The lipidbound heptasaccharide is converted to Glc_3_Man_9_GlcNAc_2_-PP-Dol by the further addition of four mannose residues from Dol-P-Man and three glucose residues from Dol-P-Glc. The tetradecasaccharide of the lipid derivative is then transferred *en bloc* to the Asn residue of the polypeptide chain, which is translated in the rough endoplasmic reticulum, by the catalytic action of a Dol-P-oligosaccharide: polypeptide oligosaccharyltransferase. Only the Asn residue in the sequence of Asn-X-Ser/Thr, where X can be any amino acid other than proline, is glycosylated. Then the completely translated protein with the tetradecasaccharide is transported to the Golgi apparatus. After the three glucose residues and four mannose residues are removed, a set of glycosyltransferases work sequentially and a variety of outer chain modifications occur.

*N*-Glycans are associated with most proteins found on the surface of mammalian cells and in secretions. *N-*Glycans bound to secreted glycoproteins have been shown to affect a wide range of protein properties such as solubility, stability, polarity, and protease sensitivity, while *N*-glycans on cell surface glycoproteins are involved in various cellular functions including cell-cell and cell-matrix interactions during embryogenesis, immune reactions, and tumor development. Therefore, defects in *N*-glycans may cause severe damage to the body. CDGs that are responsible for human diseases were initially identified in 1980. Since then, fourteen distinct CDGs have been identified.8)–11) Each is autosomal recessive and caused by mutations in different genes involved in *N*-glycosylation (Table I). Most of the disorders were discovered quite recently in one or at most a few patients. Many more types of CDG will probably be found because the ~50 genes are required for *N*-glycan synthesis. The CDGs are a group of inherited multisystemic disorders, which are commonly associated with severe psychomotor and mental retardation. CDG type I is caused by defects of the assembly of lipid-linked oligosaccharides, whereas CDG type II is caused by all defects of trimming and elongating of *N*-glycans. *N-*Glycosylation defects are routinely detected by isoelectric focusing of serum transferrin, which normally carries two-sialylated biantennary *N*-glycans. The hyposialylated transferrin from CDG patients shows a cathodic shift, which in CDG-I is due to the loss of either one or both *N-*glycans, and in CDG-II is due to the incomplete processing of protein-bound *N*-glycans. Because CDG-I results from defects in *N*-glycans that are linked to Asn residues on nascent proteins, the reduction or loss of catalytic action of a Dol-P-oligosaccharide: polypeptide oligosaccharyltransferase has been thought to be the cause of CDG-I. However, no such CDG types have been found. The molecular nature of ten CDG-I types and four CDG-II types have been identified (Table I), and several will be described here briefly.

By far the most common type of CDG, CDG-Ia [OMIM 212065, OMIM = Online Mendelian Inheritance in Man (http://www.ncbi.nih.gov/)], is caused by mutations in the *PMM2* gene. This gene encodes a phosphomannomutase that converts Man-6-P to Man-1-P. The patients were identified at first over 20 years ago based on their clinical features before the genetic basis was known. In the meantime, more than 300 patients with similar but not identical symptoms were identified. Mutations reduce the size of the GDP-Man pool and produce insufficient amount of the lipid-linked oligosaccharide for complete glycosylation. Other type-I CDGs are caused by defects in different steps of lipid-linked oligosaccharide biosynthesis. On the other hand, type-II CDGs are caused by alterations in the processing of *N-*glycans on proteins. A human disease caused by mutations in the *GnT-II* (UDP-GlcNAc: *α*-6-D-mannoside *β* - 1,2-*N*-acetylglucosaminyltransferase II) gene is known as CDG-IIa (OMIM 212066). Patients with CDG-IIa show hypotonia, severe psychomotor retardation, frequent infections, and widely spaced nipples.12) CDG-IIc (OMIM 266265), which is caused by mutations in the Golgi GDP-fucose transporter, results in fucosylation defects in the whole body, profound mental retardation, failure to thrive, recurrent infections, and leukocytosis. 13),14) CDG-IId has been observed in only one patient, who showed brain malformation, mental retardation, myopathy, and blood clotting defects. This patient was found to have a 1bp insertion in the *β 4GalT1* gene and a reduced activity of *β* 1,4galactosyltransferase.15) Taken together, the CDG studies indicate that correct *N*-glycosylation of proteins is essential for normal development.

## *O*-Mannosyl glycan and dystroglycan

*O-*Mannosylated glycoproteins are abundant in the yeast cell wall, and all *O*-mannosyl glycan structures elucidated so far are neutral linear glycans consisting of 1 to 7 mannose residues.16) *O*-Mannosylation of proteins has been shown to be vital in yeast, and its absence may affect cell wall structure and rigidity. Additionally, a deficiency in protein *O*-mannosylation in the fungal pathogen, *Candida albicans*, leads to defects in multiple cellular functions including expression of virulence. 17) In addition to fungi and yeast, clam worm has an *O*-mannosyl glycan (a glucuronyl*α*1–6mannosyl disaccharide) in skin collagen.18) Mammalian *O*-mannosylation is an unusual type of protein modification that was first identified in chondroitin sulfate proteoglycans of brain19)–21) and is present in a limited number of glycoproteins of brain, nerve, and skeletal muscle.7) In brief, we previously found that the glycans of *α*-dystroglycan include *O*-mannosyl oligosaccharides, and that a sialyl *O-*mannosyl glycan, Sia*α* 2–3Gal*β*1–4GlcNAc*β*1–2Man, is a laminin-binding ligand of *α*-dystroglycan.22) Interestingly, we found the same *O*-mannosyl glycan in rabbit skeletal muscle *α*-dystroglycan.23) After our reports of the sialylated *O*-mannosyl glycan, an HNK-1 epitope (sulfoglucuronyl lactosamine) carrying *O*-mannosyl glycan (HSO_3_-3GlcA*β*1–3Gal*β*1–4GlcNAc*β*1–2Man) was detected in total brain glycopeptides.24) It is noteworthy that these oligosaccharides have not only 2-substituted mannose but also 2,6-disubstituted mannose.25) Very recently a gene encoding this 6-branching enzyme (GnT-IX) has been cloned.26) Further, dystroglycan from sheep brain has a Gal*β*1–4(Fuc*α*1–3)GlcNAc*β*1–2Man structure27) and mouse J1/tenascin, which is involved in neuron-astrocyte adhesion, contains the *O*-mannosyl glycans.28) Therefore, it is likely that a series of *O*-mannosyl glycans, with heterogeneity of mannose-branching and peripheral structures, is present in mammals. Further studies are needed to clarify the distribution of such *O*-mannosyl glycans in various tissues and to examine their changes during development and under pathological conditions.

**Fig. 1:**
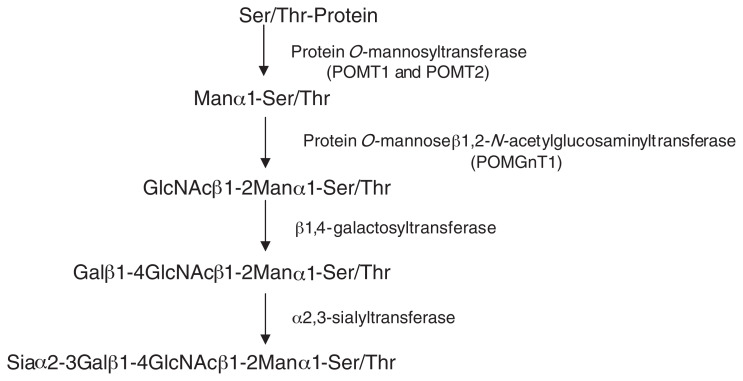
Biosynthetic pathway of mammalian *O*-mannosyl glycan.

Identification and characterization of the enzymes involved in the biosynthesis of mammalian type *O*-mannosyl glycans will help to elucidate the function and regulation of these glycans (Fig. 1). A key difference between mammalian and yeast-type *O*-mannosyl glycans is that those in mammals have the GlcNAc *β*1–2Man linkage. This linkage is assumed to be catalyzed by a glycosyltransferase, UDP-GlcNAc: protein *O*-mannose *β*1,2-*N-*acetylglucosaminyltransferase (POMGnT1). POMGnT1 catalyzes the transfer of GlcNAc from UDP-GlcNAc to *O-*mannosyl glycoproteins. We developed an enzyme assay for POMGnT1, and found its activity in several mammalian brains.29) It should be noted that GlcNAc*β*1–2Man linkages are also found in *N*-glycans, where they are catalyzed by two enzymes, UDP-GlcNAc: *α*-3-D-mannoside *β* -1,2-*N*-acetylglucosaminyl-transferase I (GnT-I) and GnT-II. However, we found that recombinant GnT-I and GnT-II had no ability to catalyze the GlcNAc *β*1–2Man linkage in *O*-mannosyl glycans,29) suggesting that a new enzyme must be responsible for the formation of this linkage. Thus, we cloned the human *POMGnT1* gene.30) The nucleotide sequence indicated that human POMGnT1 is a 660 amino acid protein and is a type II membrane protein.

Careful examination of substrate specificity of POMGnT1 indicated that POMGnT1 did not have either GnT-I or GnT-II activity.30) As described above, GnT-I and GnT-II did not have any POMGnT1 activity. Taken together, these results suggest that loss-of-function of POMGnT1 is not compensated by GnT-I and GnT-II. Mammals are known to have an absolute requirement for GnT-I during early embryogenesis. Mouse embryos lacking the functional *GnT-I* gene die prenatally at E9.5 with multisystemic abnormalities.31),32) On the other hand, over 60% of mouse embryos with null mutations in the *GnT-II* gene survive to term, but 99% of newborns die during the first week of postnatal development with multisystemic abnormalities.33) Furthermore, a human disease caused by mutations in the *GnT-II* gene is known as CDG-IIa (Table I). No human diseases having defects in *GnT-I* have been reported, suggesting that such defects result in embryonic lethality and that GnT-I is essential for normal human development.

**Fig. 2:**
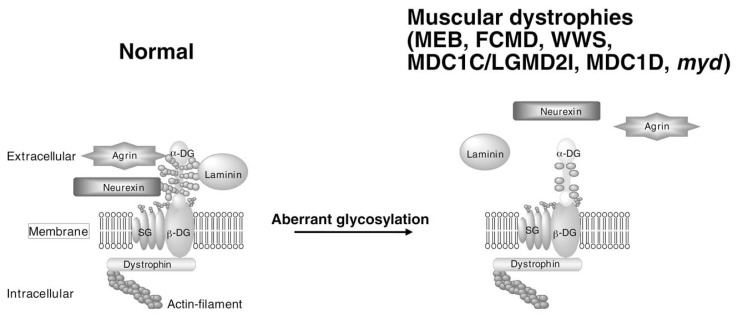
Dystrophin-glycoprotein complex (DGC) and linkage between the extracellular matrix and the subsarcolemmal cytoskeleton. Left, *α*-Dystroglycan is a key component of the DGC and is modified by *O*-mannosyl glycan and binds to laminin via its glycan. *α*-Dystroglycan is also known to bind to other extracellular matrix proteins containing laminin G-domains (LamG), such as neurexin and agrin. On the other hand, inside the cell, *β* -dystroglycan is known to bind to dystrophin and several components directly or indirectly. Right, Disruption of linkage between the extracellular components and *α*-dystroglycan due to defects of *O*-mannosyl glycan is thought to cause several muscular dystrophies (MEB, FCMD, WWS, MDC1C, LGMD2I, MDC1D and *myd* in Table III). *α*-DG, *α*- dystroglycan; *β*-DG, *β*-dystroglycan; SG, sarcoglycan.

As mentioned above, we found *O*-mannosyl glycan during structural analysis of dystroglycan glycans. Dystroglycan is encoded by a single gene (*DAG1*) and is cleaved into two proteins, *α*-dystroglycan and *β* -dystroglycan, by posttranslational processing.34),35) In skeletal muscle, dystroglycan is a central component of the dystrophin-glycoprotein complex (DGC)(Fig. 2, left). *α*-Dystroglycan is a heavily glycosylated extracellular peripheral membrane glycoprotein that anchors to the cell membrane by binding to a transmembrane glycoprotein, *β* -dystroglycan. The *α*-dystroglycan-*β* -dystroglycan complex is expressed in a broad array of tissues and is thought to stabilize the plasma membrane by acting as an axis through which the extracellular matrix is tightly linked to the cytoskeleton. This is because *α*-dystroglycan strongly binds to extracellular matrix proteins containing laminin G (LamG) domains, such as laminin, neurexin, and agrin in a calcium-dependent manner.36) On the other hand, the cytoplasmic domain of *β* -dystroglycan contains a PPXY motif that interacts with dystrophin, which in turn binds to the actin cytoskeleton. 37) Based on this molecular organization, the DGC is thought to contribute to the structural stability of the muscle cell membrane during cycles of contraction and relaxation. In human, mutations in dystrophin cause Duchenne and Becker muscular dystrophy, mutations in sarcoglycan (SG in Fig. 2) cause limb-girdle muscular dystrophy, and mutations in laminin *α* 2 chain cause congenital muscular dystrophy.38)

The function of dystroglycan in the body has been examined by targeting the *DAG1* gene in mice. However, disruption of this gene in mice results in embryonic lethality.39) To allow the embryo to develop, chimeric mice generated from targeted embryonic stem cells have been produced. Dystroglycan-null chimeric mice showed muscular dystrophy, although muscle basement membrane formation was normal.40) The function of dystroglycan in specific tissues was examined with the Cre/LoxP system. Targeting the dystroglycan gene specifically in differentiated skeletal muscle did not affect muscle basement membrane formation but resulted in a mild dystrophic phenotype.41) Targeting the dystroglycan gene in brain resulted in abnormal cerebral cortical layering resembling human cobblestone lissencephaly, and in abnormal cerebellar granule cell migration.42) Targeting the dystroglycan gene in peripheral nerves caused defects in both myelination and nodal architecture.43) These results indicate that dystroglycan is essential for normal development. As described below, not only dystroglycan itself but also the attached sugars are important.

## Muscle-eye-brain disease (MEB)

The human *POMGnT1* gene is located at 1p33, within the small candidate interval for muscle-eye-brain disease (MEB: OMIM 253280). MEB is an autosomal recessive disorder characterized by congenital muscular dystrophy, ocular abnormalities, and brain malformation (type II lissencephaly).44) Patients with MEB show severe cerebral and ocular anomalies, but some patients reach adulthood. MEB has been observed mainly in Finland.

After we screened the entire coding region and the exon/intron flanking sequences of the *POMGnT1* gene for mutations in patients with MEB, we identified 13 independent disease-causing mutations in these patients (Table II).30),45) We have not detected these 13 substitutions in any of 300 normal individuals, indicating that the mutations are pathogenic and that the *POMGnT1* gene is responsible for MEB. To confirm that the mutations observed in patients with MEB are responsible for the defects in the synthesis of *O*-mannosyl glycan, we expressed all of the mutant proteins and found that none of them had enzymatic activity.30),46) These findings indicate that MEB is inherited as a loss-offunction of the *POMGnT1* gene. If POMGnT1 does not function, no peripheral structure (Neu5Ac*α* 2–3Gal*β*1–4GlcNAc, Gal*β*1–4(Fuc*α*1–3)GlcNAc, and HSO_3_-3GlcA*β*1–3Gal*β*1–4GlcNAc) can be formed on *O*-mannose residues. Because these structures are involved in adhesive processes, a defect of *O*-mannosyl glycan may severely affect cell migration and cell adhesion. Additionally, we found a selective deficiency of *α*-dystroglycan in MEB patients.47) This finding suggests that *α*-dystroglycan is a potential target of POMGnT1 and that hypoglycosylation of *α*-dystroglycan may be a pathomechanism of MEB. MEB muscle and brain phenotypes can be explained by a loss-of-function of *α*-dystroglycan due to abnormal *O*-mannosylation.

After our report that MEB is caused by a defect of *O*-mannosylation,30) some muscular dystrophies have been suggested to be caused by abnormal glycosylation of *α*-dystroglycan, e.g., Fukuyama-type congenital muscular dystrophy (FCMD: OMIM 253800), congenital muscular dystrophy type 1C (MDC1C: OMIM 606612), Walker-Warburg syndrome (WWS: OMIM 236670), congenital muscular dystrophy type 1D (MDC1D), and the myodystrophy (*myd*) mouse (Table III).

## Walker-Warburg syndrome (WWS)

**Fig. 3:**
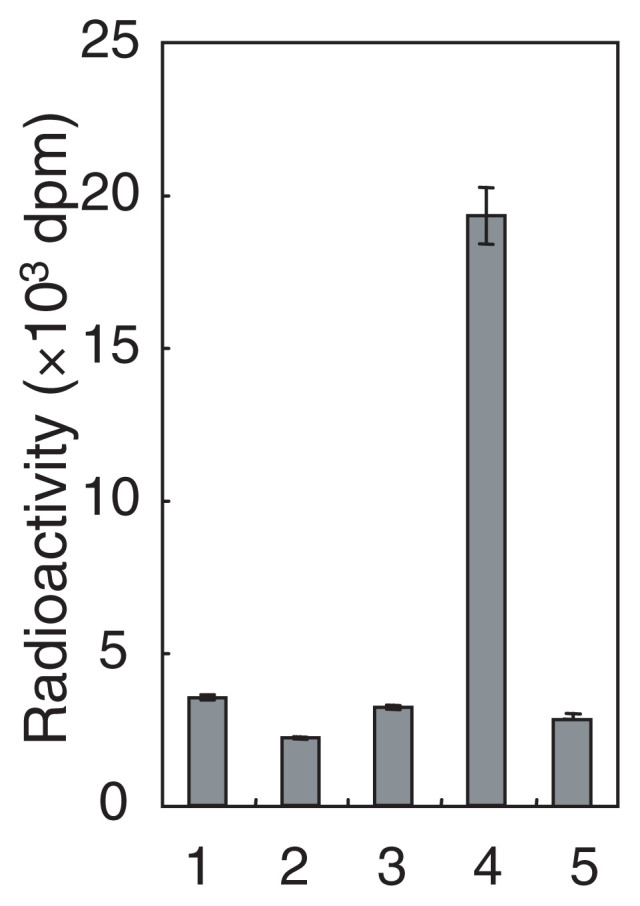
Protein *O*-mannosyltransferase activity of human POMT1 and POMT2. Protein *O*-mannosyltransferase activity of membrane fractions from HEK293T cells transfected with vector alone (1), cells transfected with human POMT1 (2), cells transfected with human POMT2 (3), cells cotransfected with POMT1 and POMT2 (4), and a mixture of the membrane fractions from *POMT1*-transfected cells and *POMT2*-transfected cells (5). Reprinted with permission from ref. 51). Copyright (2004) National Academy of Sciences, U.S.A.

WWS is another form of congenital muscular dystrophy that is characterized by severe brain malformation (type II lissencephaly) and eye anomalies.48) Patients with WWS are severely affected from birth and usually die within their first year. WWS has a worldwide distribution. Recently, 20% of WWS patients (6 of 30 unrelated WWS cases) have been found to have mutations in protein *O*-mannosyltransferase 1 (*POMT1*), a putative *O-*mannosyltransferase that catalyzes the transfer of mannose to a Ser or Thr residue on the basis of homology with seven yeast protein *O*-mannosyltransferases.49) *POMT1* is highly expressed in fetal brain, testis, and skeletal muscle, which are the affected tissues in WWS. It is noteworthy that none of the 30 cases studied had mutations in another homologue, *POMT2*, which is 33% identical to *POMT1*. However, it was unclear whether the POMT1 and POMT2 proteins actually catalyze protein *O-*mannosylation, 49),50) and attempts to detect protein *O-*mannosyltransferase activity of POMTs in vertebrates have not been successful. Recently, we developed a new method to detect the enzymatic activity of protein *O-*mannosyltransferase in mammalian cells and tissues. Using this new method, we demonstrated that human POMT1 and POMT2 have protein *O*-mannosyltransferase activity, but only when they are co-expressed (Fig. 3).51) This suggests that POMT1 and POMT2 form a hetero-complex to express enzymatic activity. *POMT1* and *POMT2* are expressed in all human tissues, but *POMT1* is highly expressed in fetal brain, testis, and skeletal muscle, and *POMT2* is predominantly expressed in testis.50),52) *O*-Mannosylation seems to be uncommon in mammals and only a few *O*-mannosylated proteins have been identified.7) It will be of interest to determine the regulatory mechanisms for protein *O*-mannosylation in each tissue. In view of the potential importance of this form of glycosylation for a number of developmental and neurobiological processes, the ability to assay vertebrate *O*-mannosyltransferase activity and knowledge of the requirement of a heterodimeric complex for enzyme activity should greatly facilitate progress in the identification and localization of *O*-mannosylated proteins and the elucidation of their functional roles.

Recently, 6 of 30 WWS patients were found to have mutations in *POMT1*, while none had mutations in *POMT2*.49) A possible explanation for the absence of *POMT2* mutations in human subjects is that *POMT2* may be essential for normal development, i.e., *POMT2* mutations may result in embryonic lethality. Another possibility is that patients with *POMT2* mutations were simply not included in the 30 WWS patients. A worldwide survey of the occurrence of *POMT2* mutations is needed to determine whether WWS is caused by *POMT* mutations.

In WWS patients, as in MEB patients, the glycosylated *α*-dystroglycan was selectively deficient in skeletal muscle. WWS and MEB are clinically similar autosomal recessive disorders that are characterized by congenital muscular dystrophy, lissencephaly, and eye anomalies, but WWS is a more severe syndrome than MEB.48),53) Patients with WWS are severely affected from birth (brain malformation is particularly common), and few live beyond infancy. In MEB, the cerebral and ocular anomalies are also severe, but some patients reach adulthood.44),53) The difference of severity between the two diseases may be explained as follows: If POMGnT1, which is responsible for the formation of the GlcNAc*β*1–2Man linkage of *O*-mannosyl glycans,30) is non-functional, only *O*-mannose residues may be present on *α*-dystroglycan in MEB. On the other hand, *POMT1* mutations cause complete loss of *O*-mannosyl glycans in WWS. It is possible that attachment of a single mannose residue on *α*-dystroglycan is responsible for the difference in clinical severity of WWS and MEB.

Interestingly, the *Drosophila rt* mutant exhibiting defects of myogenesis was found to be due to a mutation in a homologue of *POMT1*.50),54) The mutation also causes reduced fertility and reduced viability. Although the *rt* gene product is not known to be a component involved in the initial step of *O*-mannosyl glycan biosynthesis, *O-*mannosylation is an evolutionarily conserved protein modification,7) and may be essential for muscle development in both vertebrates and invertebrates.

## Fukuyama-type congenital muscular dystrophy (FCMD)

Like MEB and WWS, FCMD is an autosomal recessive disorder that is characterized by congenital muscular dystrophy, lissencephaly, and eye anomalies and FCMD is a relatively common autosomal recessive disorder in the Japanese population.55) It is the second most common form of childhood muscular dystrophy in Japan after Duchenne muscular dystrophy. Based on an average incidence of 3 per 100,000 population, one in ~90 persons could be a heterozygous carrier in Japan. Kobayashi *et al*.56) identified a gene on chromosome 9q31 that is responsible for FCMD. The gene encodes a novel 461 amino acid protein of unknown function. The protein, named fukutin because of its association with FCMD, has an N-terminal hydrophobic region which would be a signal sequence or a transmembrane domain. A sequence analysis predicts that it could be an enzyme involved in glycosylation.57) Consistent with this finding, highly glycosylated *α*-dystroglycan was selectively deficient in the skeletal muscle of FCMD patients.58) Recently, Takeda *et al*.59) generated chimeric mice using embryonic stem cells in which the *fukutin* gene was targeted for disruption. These mice developed severe muscular dystrophy, with a selective deficiency of *α*-dystroglycan and its laminin-binding activity. These mice also had central nervous and ocular abnormalities. Taken together, these results indicate that fukutin is necessary for the maintenance of muscle integrity, cortical histogenesis, and normal ocular development, and suggest a functional linkage between fukutin and *α*-dystroglycan.

## Congenital muscular dystrophy type 1C (MDC1C) and limb-girdle muscular dystrophy 2I (LGMD2I)

Defective glycosylation of *α*-dystroglycan has also been implicated in congenital muscular dystrophy type 1C (MDC1C), which is caused by a homologue of *fukutin* (fukutin-related protein, *FKRP*).60) MDC1C is characterized by severe muscle weakness and degeneration, and cardiomyopathy. Mental retardation and cerebellar cysts have been observed in some cases. Allelic mutations in the *FKRP* gene also cause a milder and more common form of muscular dystrophy called limb-girdle muscular dystrophy 2I (LGMD2I: OMIM 607155), which is frequently associated with cardiomyopathy and shows variable onsets ranging from adolescence to adulthood.61) Patients with the mutations in the *FKRP* gene invariably exhibit a reduced expression of *α*- dystroglycan, which is strongly correlated with disease severity. A western blot analysis showed an apparent loss of higher molecular weight forms of *α*-dystroglycan. Although the function of FKRP is unknown, it has been suggested that FKRP is involved in the glycosylation of *α*-dystroglycan as a glycosyltransferase or a kind of modulator. Because FKRP and fukutin are thought to be Golgi-resident proteins,62) it is possible that defects of these proteins cause abnormal processing of *α*-dystroglycan.

## Congenital muscular dystrophy type 1D (MDC1D) and the myodystrophy (*myd*) mouse

The gene *large*, which is mutated in the myodystrophy (*myd*) mouse, encodes a putative glycosyltransferase. 63) However, its biochemical activity has not been confirmed. The causative mutation in *myd* was identified as a deletion of exons 5–7 of the *large* gene. This deletion results in a frameshift in the corresponding mRNA, leading to a premature termination codon. The *myd* mouse shows a progressive muscular dystrophy, ocular defects, and a central nervous system phenotype characterized by abnormal neuronal migration in the cerebral cortex, cerebellum, and hippocampus, and disruption of the basal lamina.64),65) The *myd* mouse, like MEB and FCMD patients, showed hypoglycosylation of *α*-dystroglycan in muscle and brain. The human homologue of the *large* gene (*LARGE*) may be involved in novel forms of muscular dystrophy. A recent study66) described a patient with congenital muscular dystrophy, profound mental retardation, white matter changes, and subtle structural abnormalities in the brain and a reduction of immunolabelling of *α*-dystroglycan. This type of muscular dystrophy was named as MDC1D. The patient was found to have a missense mutation and a 1bp insertion in the *LARGE* gene.

## Perspectives

Unlike proteins and nucleic acids, which are linear molecules, sugar chains form branching, and positional and anomeric isomers, indicating the occurrence of a remarkable number of structures with a small number of units. Such complexity has made their structural analysis difficult and has obscured their functions for a long time. However, newly developed and sensitive methods to elucidate the structures and functions of the sugar chains have made it possible to precisely determine small amounts of sugar chains. Such studies have shown that glycans are highly abundant and exhibit diverse structures, with widely varying functions. Newly available genetic approaches accelerate discoveries of these functions. Furthermore, many examples of genetic alterations in glycan structures and expression have been found in humans, and have provided many clues to glycan functions. In the future, progress in understanding glycan functions will continue to rely on glycan structural analyses based on mutational analyses. Because the amount of material is often limited, it is essential to develop more sensitive methods for analyzing the structures of the sugar chains.

**Fig. 4:**
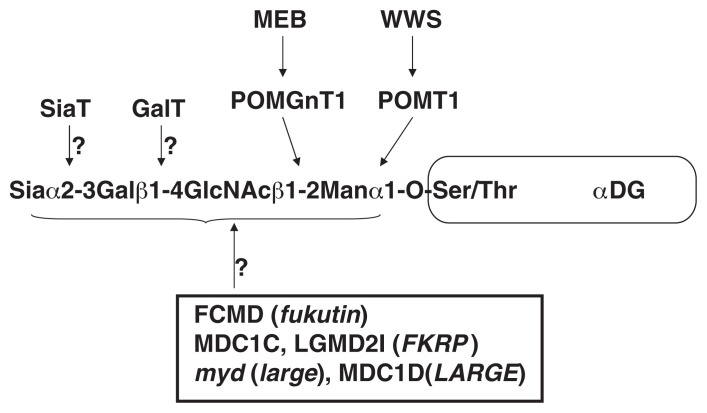
Possible defects of *O*-mannosylglycosylation of *α*-dystroglycan in muscular dystrophy. Mutations in *POMGnT1*, *POMT1*, *fukutin*, *FKRP* and *LARGE* (*large*) cause defects in the glycosylation of *α*-dystroglycan resulting in muscular dystrophy. The substrates of these putative enzymes, with the exception of POMGnT1 and POMT1, are largely unknown. It is unclear whether other as yet uncharacterized forms of muscular dystrophy are caused by defects in galactosyltransferases (GalT) and sialyltransferases (SiaT).

*O*-Mannosylation is an unusual type of protein modification and is present in a limited number of glycoproteins of brain, nerve, and skeletal muscle. *O-*Mannosyl glycans play critical roles in the following example. Hypoglycosylated *α*-dystroglycan, which is probably caused by a defect of *O*-mannosylation, has greatly reduced affinities for laminin, neurexin and agrin.67) This suggests that defective glycosylation of *α*-dystroglycan due to the genetic defects of glycosyltransferases is the common trait of muscle cell degeneration and abnormal brain structure found in MEB, WWS, FCMD, MDC1C, MDC1D patients and the *myd* mouse (Fig. 2). Therefore, *α*-dystroglycan may be a potential target of future therapy for muscular dystrophy. However, the substrates of these enzymes (Table III), with the exception of POMGnT1 and POMT1, are largely unknown (Fig. 4). Identification and characterization of each enzyme will help to reveal the molecular pathomechanisms of congenital muscular dystrophies with brain malformation. Future studies may also reveal that presently uncharacterized forms of muscular dystrophy are caused by defects in galactosyltransferases and/or sialyltransferases. A major challenge will be to integrate the forthcoming structural, cell biological, and genetic information to understand how *α*-dystroglycan glycosylation contributes to muscular dystrophy.

## Figures and Tables

**Table I:** Congenital disorders of glycosylation (CDGs)

CDG type	Gene	Protein function	Gene locus
Ia	*PMM2*	Phosphomannomutase 2	16p13.3-p13.2
Ib	*MPI*	Phosphomannose isomerase	15q22-qter
Ic	*ALG6*	Dol-P-Glc: Man_9_GlcNAc_2_-PP-Dol *α*1,3glucosyltransferase	1p22.3
Id	*ALG3*	Dol-P-Man: Man_5_GlcNAc_2_-PP-Dol *α*1,3mannosyltransferase	3q27
Ie	*DPM1*	Dol-P-Man synthase 1	–
If	*MPDU1*	Dol-P-Man utilization defect 1	–
Ig	*ALG12*	Dol-P-Man: Man_7_GlcNAc_2_-PP-Dol *α*1,2mannosyltransferase	22
Ih	*ALG8*	Dol-P-Glc: Glc_1_Man_9_GlcNAc_2_-PP-Dol *α*1,3glucosyltransferase	–
Ii	*ALG2*	GDP-Man: Man_1_GlcNAc_2_-PP-Dol *α*1,3mannosyltransferase	9q22
Ij	*DPAGT1*	UDP-GlcNAc: Dol-P-GlcNAc phosphotransferase	11q23.3
IIa	*MGAT2*	UDP-GlcNAc: *α*-6-mannoside *β*1,2 *N*-acetylglucosaminyltransferase (GnT-II)	14q21
IIb	*GCS1*	*α*1,2glucosidase I	2p13-p12
IIc	*FUCT1*	GDP-fucose transporter	11
IId	*B4GALT1*	UDP-Gal: *N*-acetylglucosamine *β*1,4galactosyltransferase (GalT-1)	9q13

**Table II:** Summary of mutations found in patients with MEB

	Mutation		Effect
1	281 C > T		Arg63Stop Nonsense
2	541 del T		Phe149 frameshift 167Stop
3	761 G > A		Glu223 Lys Missense
4	900 G > A		Cys269 Tyr Missense
5	1077 ins G		Val328 frameshift 338Stop
6	1106 ins T		Asp338 frameshift 338Stop
7	1572 C > G		Pro493 Arg Missense
8	IVS17+1 G > A	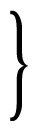	Glu514 read-through 526Stop
9	IVS17+1 G > T		and Leu472-His513 del
10	1743 G > A		Ser536-Ser550 del
11	1813 del C		His573 frameshift 633Stop
12	1926 del T		Leu611 frameshift 633Stop
13	1970 del G		Val626 frameshift 633Stop

**Table III:** Possible muscular dystrophies caused by abnormal glycosylation of *α*-dystroglycan

Condition	Gene	Protein function	Gene locus
Muscle-eye-brain disease (MEB)	*POMGnT1*	GlcNActransferase	1p33
Fukuyama-type congenital muscular dystrophy (FCMD)	*fukutin*	Putative glycosyltransferase	9q31
Walker-Warburg syndrome (WWS) (20%)	*POMT1*	*O*-Mannosyltransferase	9q34.1
MDC1C and limb-girdle muscular dystrophy 2I(LGMD2I)	*FKRP* (fukutin-related protein)	Putative glycosyltransferase	19q13.3
Myodystrophy (*myd*) mouse MDC1D	*large* *LARGE*	Putative glycosyltransferase	8 (mouse) 22q12.3-13.1
